# A transcriptome-based classifier to determine molecular subtypes in medulloblastoma

**DOI:** 10.1371/journal.pcbi.1008263

**Published:** 2020-10-29

**Authors:** Komal S. Rathi, Sherjeel Arif, Mateusz Koptyra, Ammar S. Naqvi, Deanne M. Taylor, Phillip B. Storm, Adam C. Resnick, Jo Lynne Rokita, Pichai Raman

**Affiliations:** 1 Center for Data-Driven Discovery in Biomedicine, Children’s Hospital of Philadelphia, Philadelphia, Pennsylvania, United States of America; 2 Department of Biomedical and Health Informatics, Children’s Hospital of Philadelphia, Philadelphia, Pennsylvania, United States of America; 3 Division of Neurosurgery, Children’s Hospital of Philadelphia, Philadelphia, Pennsylvania, United States of America; 4 Department of Pediatrics, Perelman School of Medicine, University of Pennsylvania, Philadelphia, Pennsylvania, United States of America; Johns Hopkins University, UNITED STATES

## Abstract

Medulloblastoma is a highly heterogeneous pediatric brain tumor with five molecular subtypes, Sonic Hedgehog *TP53*-mutant, Sonic Hedgehog *TP53*-wildtype, WNT, Group 3, and Group 4, defined by the World Health Organization. The current mechanism for classification into these molecular subtypes is through the use of immunostaining, methylation, and/or genetics. We surveyed the literature and identified a number of RNA-Seq and microarray datasets in order to develop, train, test, and validate a robust classifier to identify medulloblastoma molecular subtypes through the use of transcriptomic profiling data. We have developed a GPL-3 licensed R package and a Shiny Application to enable users to quickly and robustly classify medulloblastoma samples using transcriptomic data. The classifier utilizes a large composite microarray dataset (15 individual datasets), an individual microarray study, and an RNA-Seq dataset, using gene ratios instead of gene expression measures as features for the model. Discriminating features were identified using the limma R package and samples were classified using an unweighted mean of normalized scores. We utilized two training datasets and applied the classifier in 15 separate datasets. We observed a minimum accuracy of 85.71% in the smallest dataset and a maximum of 100% accuracy in four datasets with an overall median accuracy of 97.8% across the 15 datasets, with the majority of misclassification occurring between the heterogeneous Group 3 and Group 4 subtypes. We anticipate this medulloblastoma transcriptomic subtype classifier will be broadly applicable to the cancer research and clinical communities.

This is a *PLOS Computational Biology* Software paper.

## Introduction

Medulloblastoma (MB) is the most common of childhood brain tumors and accounts for nearly 20% of all pediatric CNS neoplasms [1]. Based on histology, these tumors are classified as high-grade embryonal tumors, but they comprise of molecularly distinct subtypes based on their genetics [2]. The current long-term survival rate is approximately 70%, however there is significant variation in survival in the population based on molecular subtype, age, residual disease, presentation of metastases, and other factors [3]. Additionally, conventional treatments can cause significant morbidity in patients with several long-term consequences [4]. This prompted the medical and research communities to seek other treatment options that are more targeted, engendering a focus on molecular characterizations of these tumors. In 2016, based on several profiling studies, five molecular subtypes of MB were recognized, Sonic HedgeHog (SHH) *TP53* mutant, SHH *TP53* wild-type, WNT, Group 3, and Group 4 [5,6]. Specifically, these subtypes were independently identified and demonstrated as concordant from multiple bioinformatic analyses of gene expression, comparative genomic hybridization, and DNA methylation microarray data: prediction analysis of microarrays [7], unsupervised two-way hierarchical clustering and bootstrap analysis [8], unsupervised SubMap [7], non-negative matrix factorization [7,9]. These subtypes are widely used in both the research and clinical communities and have been accepted by the World Health Organization [6]. Currently, clinical classification of MB is most frequently accomplished by immunostaining, but genomic methods, specifically, methylation profiling [10,11], have recently supplemented immunostaining in the clinic. The use of methylation over gene expression microarrays or RNA-Sequencing (RNA-Seq) has predominated because RNA tends to degrade at a much faster rate relative to DNA or methylation markers [9] and historically, methylation arrays were more cost effective than RNA-Seq. However, there are several reasons to develop a classifier based on transcriptomic data. First, there are numerous studies and experiments that have deposited MB transcriptomic data without the corresponding molecular subtypes listed. Being able to quickly classify these samples into molecular subtypes may help with retrospective and/or new analysis of these studies. Second, when MB RNA-Seq data is generated without corresponding methylation data, this classifier will enable investigators to subtype their samples. Third, the prediction output of this tool may be used in conjunction with predictions from other modalities such as immunostaining, methylation, or genetic data to confirm classification of samples that may classify as more than one molecular subtype. Fourth, even though MB subgrouping has increased in complexity, clinical practice often still relies on the SHH, WNT and non-SHH/WNT subtyping only [5,12,13], and here, we show that gene expression classification enables this type of subgrouping reliably. RNA-Seq has substantial benefits compared to gene expression microarrays in both the ability to capture a much more exhaustive dynamic range and to quantify many more genes and/or isoforms [14].

We sought to develop a tool that can accurately predict the four major molecular subtypes of medulloblastoma, SHH, WNT, Group 3, and Group 4 using any type of transcriptomic data, including RNA-Seq, microarray data, or panel data from NanoString nCounter or HTG platforms. Frequently, classifiers work with one type of technology, but cannot be used outside of that platform. The classifier we have developed can span technologies because we don’t rely on individual gene expression measures and instead use gene expression ratios (GER). The use of GERs is not novel and has been utilized previously for other disease subtyping and classification efforts [15–17]. The major draw-back of using GERs is that the number of features is far greater than using individual gene measures and presents a tremendous problem from a feature selection perspective, which we mitigate here by filtering genes on low expression and low variance as well as by only using subtype specific upregulated genes as input for the GER matrix. The most significant benefits include having more features to choose from, corresponding to interesting gene regulatory networks, and having features with a level of self-normalization that result from calculating a ratio. Thus, the use of GERs, in addition to the classification method, allows us to use the same classifier regardless of how the transcriptomic data was generated, assuming that standard initial transformations & normalizations have been applied e.g. FPKM for RNA-Seq and RMA for microarray.

We tested the classifier across a number of datasets to show its predictive power and have developed both a package (https://github.com/d3b-center/medullo-classifier-package) and web application (code: https://github.com/d3b-center/medullo-classifier-shinyapp, portal: https://komalrathi.shinyapps.io/medulloclassifiershinyapp) that can be used to classify transcriptomic data. It is important to note that there are recent studies that classify medulloblastomas into many more subgroups utilizing methylation data [11, 18], however, there is insufficient training data, that is, patient tumors with transcriptomic data labeled using these subgroups, to develop a model to classify into these more granular subtypes. Therefore, at the current time, we can only capture the four main subtypes of MB [11].

## Design and implementation

### Classifier development

The Medulloblastoma subtype classifier was trained and developed on two different transcriptomic datasets using different platforms and from different research groups. The first dataset profiled 97 primary medulloblastoma samples using RNA-Seq (European Genome-Phenome Archive (EGA) accession number EGAD00001001899). Data was processed and normalized similar to [19]. The second dataset was a microarray dataset characterized using the Affymetrix Human Genome U133 Plus 2.0 Array comprised of 76 primary medulloblastoma samples (Gene Expression Omnibus (GEO) accession number GSE37418). Both datasets had corresponding molecular subtype labels, though some programmatic edits were required to harmonize them.

**Fig 1:**
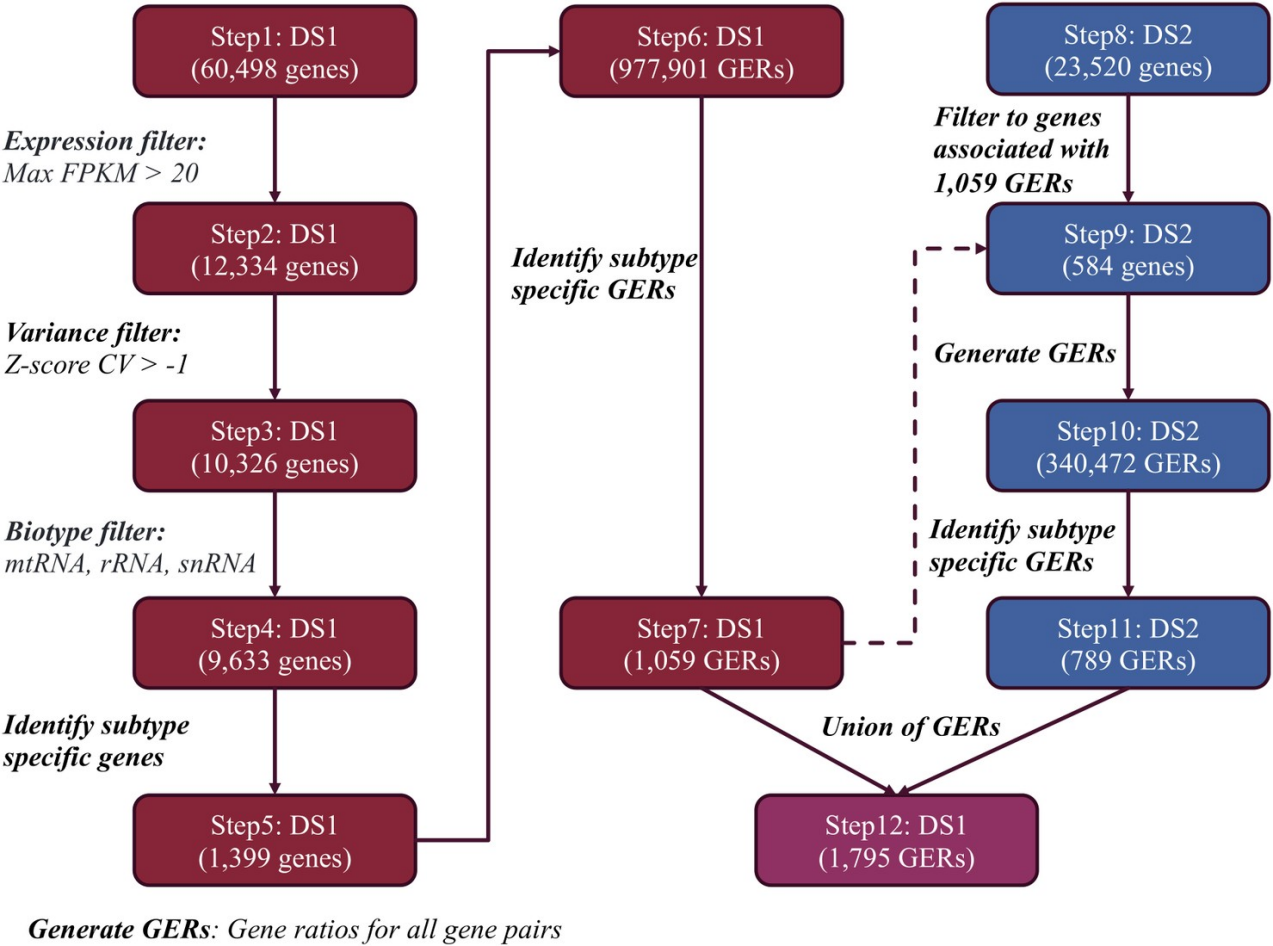
Medulloblastoma subtype-specific feature selection workflow. Workflow of identifying MB subtype-specific GERs using two transcriptomic data sets. *DS1* represents the RNA-seq dataset EGAD00001001899 and *DS2* represents the Microarray dataset GSE37418.

**Fig 1** depicts the workflow for choosing GERs for each medulloblastoma subtype. The RNA-Seq dataset was first filtered for genes for which at least one sample had an FPKM > 20. This was done to ensure expression was sufficiently high enough that variance in the gene expression was not driven by technical artifacts and noise (**S1A Fig**). The expression matrix was next log-transformed, Z-scored, and filtered at a threshold of -1 coefficient of variation (CV). This cut-off was determined based on examination of the distribution of CVs for all genes in the dataset (**S1B Fig**). Finally, all genes corresponding to mitochondrial, ribosomal, and small nuclear RNA genes were excluded, as they have varied and often-times very high expression depending on sampling and capture methodology. The filtering steps were necessary in order to reduce the number of genes since the size of GERs grows dramatically as the number of genes increases. The filtered expression matrix was then analyzed to identify genes specific to each subtype, both upregulated and downregulated. Specifically, differential expression to select gene expression features was performed in three steps. In step 1, we used variable logFC as a cutoff + adj p.value < 0.05 and gathered unique subtype-specific up/down regulated genes, that is, genes differentially expressed in one group versus all other groups. This corresponded to taking the intersection of the differentially expressed genes for any given subtype versus the other groups. In step 2, we gathered the top 250 differentially expressed genes, sorted by adj. p-value, for each pairwise comparison per subtype and took a union of these genes. While step 1 captured features specific to a given subtype, step 2 allowed us to capture features highly discriminatory between any two subtypes, but not necessarily specific to one subtype. In both steps we observed recurrent features that were discriminatory for the different subtypes and comparisons. For example, in step 1, *PTCH2* was upregulated in SHH versus other subtypes, but downregulated in WNT compared to other subtypes. In step 2, *MMP2* was upregulated in SHH vs Group 4 but also upregulated in WNT vs Group 3 and WNT vs Group 4. In the final step, step 3, we pooled the results of step 1 and step 2 and then selected genes that were both significantly upregulated in at least one subtype and significantly downregulated in another. At the end of these three steps, we obtained 1,399 genes, which were used as input for t-SNE and heatmap clustering of the subtypes. Next, a GER matrix was created from the 1,399 genes and limma was performed to obtain subtype-specific upregulated genes using the limma R package [20] with a Benjamini-Hochberg (BH) adjusted p-value cut-off of 0.05 and variable log-fold change thresholds. This filtered set of genes served as input to the next step of the pipeline, the creation of subtype-specific GERs. In this step, ratios for all pairs of genes from the filtered gene set were calculated. These were then analyzed to search for GERs specific to certain subtypes using the limma R package. The outputs of the RNA-Seq analysis were four sets of GERs, each specific to a medulloblastoma molecular subtype. Subsequent to this, a microarray expression matrix (GSE37418) was filtered to genes that were part of the GERs obtained from the RNA-Seq dataset. This expression matrix was then converted from a gene matrix to a GER matrix and the limma R package was used to identify GERs specific to each subtype based on this dataset. The GERs isolated from this microarray dataset were finally merged with those from the RNA-Seq analysis to create the model used for classification of all subsequent studies.

### Classifier evaluation

To test the accuracy of the classifier, we chose a superset of 23 GEO datasets containing 1,641 samples (GEO accession id: GSE124814). From the 23 datasets, we removed one dataset that was used for training the classifier, GSE37418 (n = 95) and removed seven datasets which did not have subtype information associated with them: GSE22569, GSE25219, GSE3526, GSE35974, GSE4036, GSE44971, GSE60862. The classifier was then evaluated on the remaining 15 medulloblastoma datasets (N = 1,286 samples) with both expression data and corresponding molecular subtype classification data.

The classification steps for each of these datasets were 1) convert the gene-expression matrix into a GER matrix, 2) filter to GERs in the model, 3) take the mean-score of GERs for each subtype, and 4) assign the sample with the subtype corresponding to the highest score (taking an unweighted mean of normalized scores) [20]. We also performed a t-test of the best predicted subtype vs. the remaining three subtypes and took the maximum p-value representing a confidence score associated with the predicted subtype. This step was employed because, as the classification system is coded, a class will always be selected. However, it is probable that for certain samples two or more classes may have scores not significantly different from one another, raising the likelihood of a misclassification. This p-value indicates if the mean GERs of the selected class is significantly higher than the mean GER score of the class with the second highest mean GER. A very low p-value should not be interpreted as related to the probability of that classification being correct. However, a high p-value may warrant a deeper look at the data to verify the prediction. Accuracy, precision, and evaluation metrics were obtained by using the confusionMatrix function from the caret R package [21]. Finally, for the 15 test datasets, we compared the performance of our medulloblastoma classifier to the previously published gene expression *Medullo-Model To Subtype* (MM2S) classifier [22,23], which performs k-nearest neighbor classification using a single sample gene-set enrichment analysis ranked matrix. Summary plots were generated using the ggplot package.

### Package & portal creation

We developed an R package, *medulloClassifierPackage*, to facilitate annotation of user-supplied medulloblastoma samples and transcriptomic datasets with molecular subtype information. The package consists of 2 functions, ***Classify*.*R*** and ***calcStats*.*R***. The ***Classify*** function takes in a sample expression dataset and returns back a character vector of the classes & a relative confidence metric associated with each class. The confidence metric is derived from calculating T-statistics between the predicted class and the other classes from GER scores and then assigning a prediction using the max p-value. This corresponds to comparing the predicted class, the class with the highest GER average, with the class with the second highest score. Users may use this as a threshold to reject or accept the given prediction to control for sensitivity and specificity for their given project. The second function, ***calcStats***, takes in a sample expression dataset and user-supplied classes and returns statistics related to how well the RNA-Seq model-based prediction matches the classes provided by the user. This will help validate the classifier for in-house use and/or may be used to compare against other predictors to identify more robust classification models. The R Package contains a *README* file to provide the user with a descriptive overview of the package including the project objectives, methods, and installation details. In addition to the package, we have developed a Shiny Portal that allows users to upload a file corresponding to a medulloblastoma expression datasets via a browser and ascertain a list of subtype predictions.

## Results

**Fig 2:**
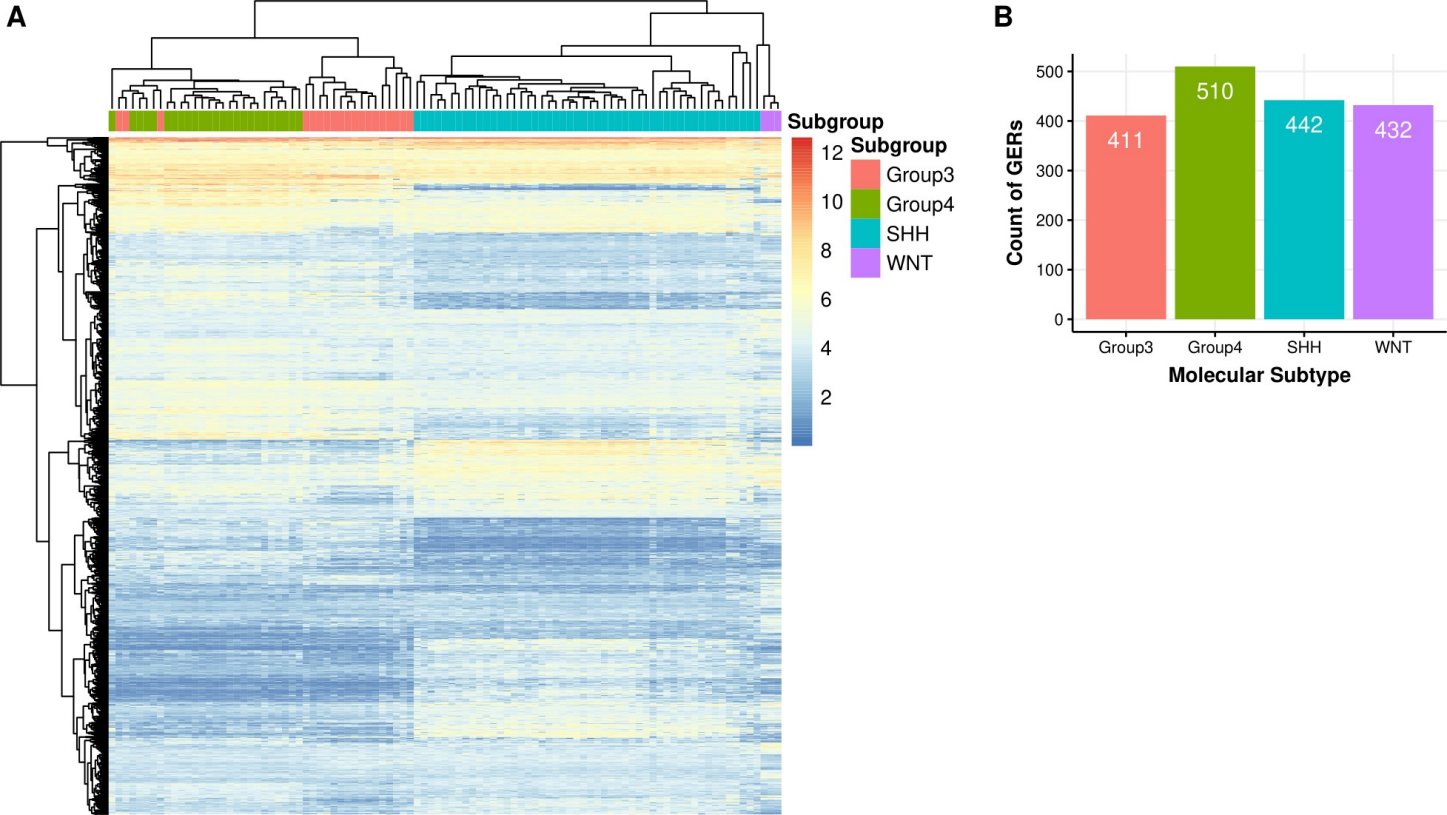
Top genes and GERs representing each Medulloblastoma subtype. **A.** Heatmap of 1,399 genes associated with 4 molecular subtypes across 97 primary MB samples (RNA-seq: EGAD00001001899). **B.** Bar chart showing count of 1,795 selected GERs associated with each MB molecular subtype.

Our final filtered list (see above for methods) maximizing separation, resulted in a final list of 1,399 genes (**S1 Table**). These select genes were able to separate the DS1 into specific subgroups as shown in the clustered heatmap (**Fig 2A**).

**Fig 3:**
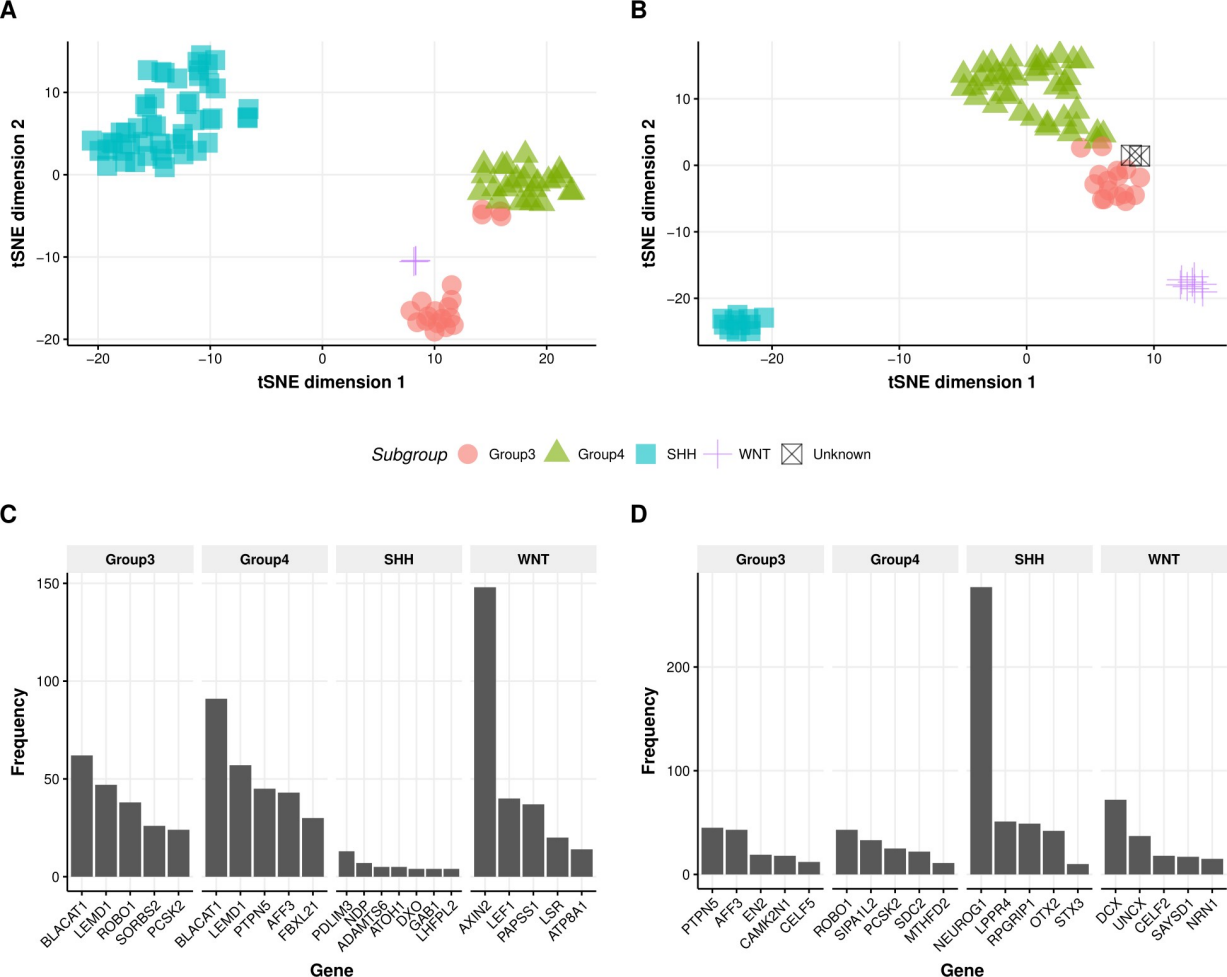
Selected GERs are able to distinguish between the four Medulloblastoma subtypes. **A.** t-SNE Plot of 1,795 selected GERs across 97 primary medulloblastoma **(**RNA-seq: EGAD00001001899). **B.** t-SNE Plot of 1,795 selected GERs across 76 primary medulloblastoma **(**Microarray: GSE37418). **C.** Bar chart showing top 5 genes frequently occuring in numerator of GERs in each molecular subtype. **D.** Bar chart showing top 5 genes frequently occuring in denominator of GERs in each molecular subtype.

**Fig 4:**
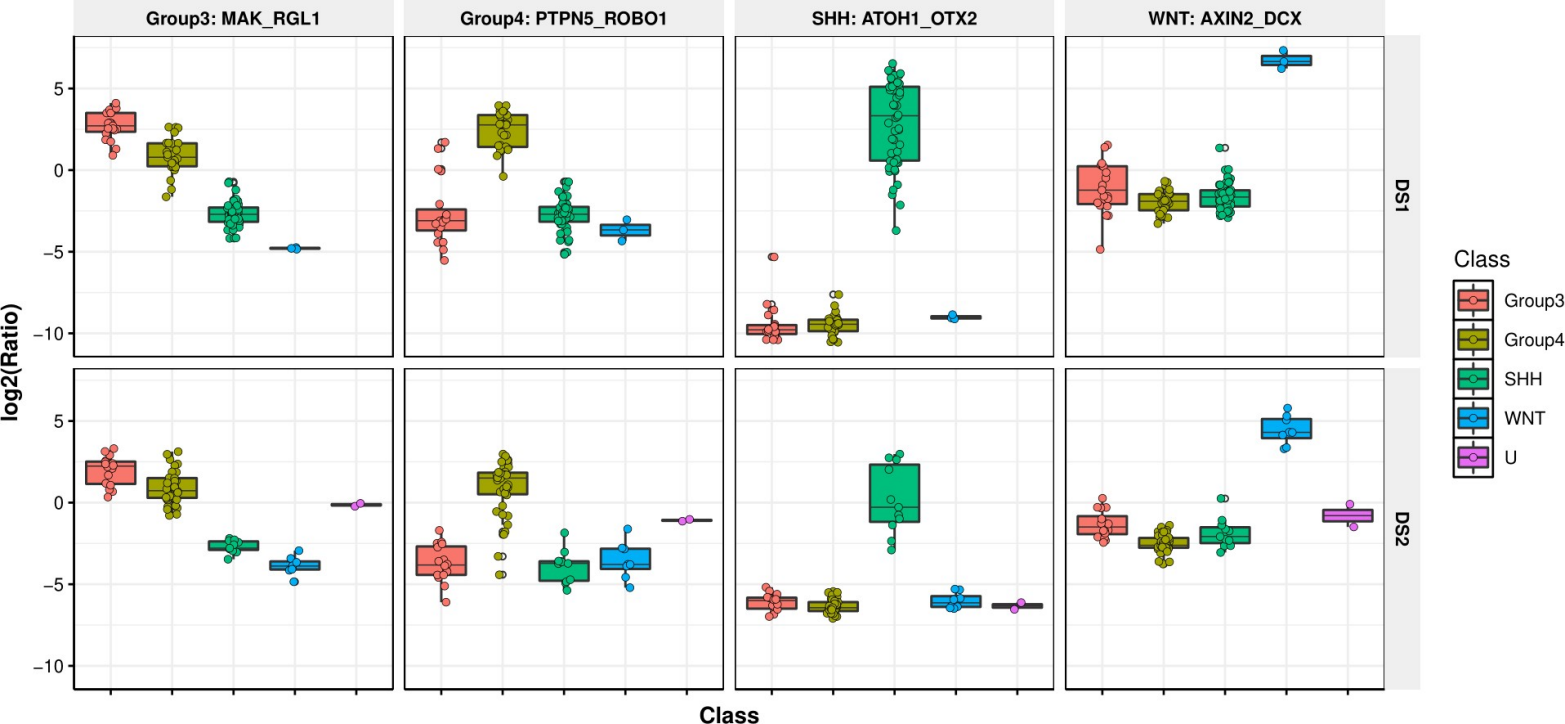
GERs associated with SHH and WNT are more discriminatory compared to Group 3 and Group 4 subtypes. Boxplot of example GERs associated with molecular subtypes across 2 studies. *DS1* represents the RNA-seq dataset EGAD00001001899 and *DS2* represents the Microarray dataset GSE37418.

These 1,399 genes were then used to generate 977,901 GERs which were filtered to identify GERs specific to each subtype. In this step, we only chose GERs that were either significantly upregulated in a single subtype in comparison to all others, resulting in a final list of 1,059 GERs corresponding to 609 genes. These genes were used to initially filter DS2 (overlap of which ended in only 584 genes) at which point GERs were calculated, resulting in a total set of 340,472 GERs. Filtering for GERs that were significantly upregulated in one subtype, and not in the others, a final set of 789 GERs from DS2 were identified. The union of GERs from DS1 and DS2, resulted in a set of 1,795 GERs (**S2 Table**). A breakdown is shown in **Fig 2B**. Surprisingly, the overlap between the significant GERs between DS1 and DS2 was smaller than anticipated, especially given that the initial sets of genes were identical. We thus wanted to confirm that the union of the GERs was still able to split up these datasets into their molecular subtypes. For both datasets, we filtered to the 1,795 GERs and then performed t-SNE analysis using the R package Rtsne using the following parameters: initial dimensions to be retained in the initial PCA step i.e. initial_dims set to 200, perplexity set to 10 and maximum iterations i.e. max_iter set to 500. We utilized t-SNE to determine if the samples clustered into distinct groups and indeed observed that molecular subtypes in both datasets were able to be captured by this union set of GERs, with the RNA-Seq dataset able to better separate Group 3 and 4 subtypes than the microarray dataset, as shown in **Fig 3A & 3B**. Interestingly, we observed four cases in the RNA-Seq dataset which were classified pathologically as the Group 3 subtype, but clustering based on the GERs selected, these tumors are potentially the Group 4 subtype (**Fig 3A**), demonstrating the utility of this classifier even when orthogonal methods are being used to determine medulloblastoma subtypes. Though our primary goal was to develop a medulloblastoma classifier based on RNA-Seq data, we were curious as to the GERs associated with each molecular subtype. In order to determine this, we assessed genes from each of the GERs and plotted their frequency in each molecular subtype. The top five GERs distinguishing each subtype are shown in **Fig 3C & 3D**, and include expected pathway driver genes. The GERs are specific to each subtype in that they have markedly higher expression in that subtype versus other subtypes. Classification was performed by calculating the mean GER score for each class and then identifying the class with the maximum mean GER score. Since the GERs are all ratios, they are approximately normalized and hence are comparable without any other type of additional transform **(S2A Fig & S2B Fig)**. Example GERs for each subtype in DS1 and DS2 are shown in **Fig 4**. The GERs associated with SHH and WNT are much more discriminatory than those for Group 3 and Group 4 subtypes, the latter of which are more transcriptomically similar.

**Fig 5:**
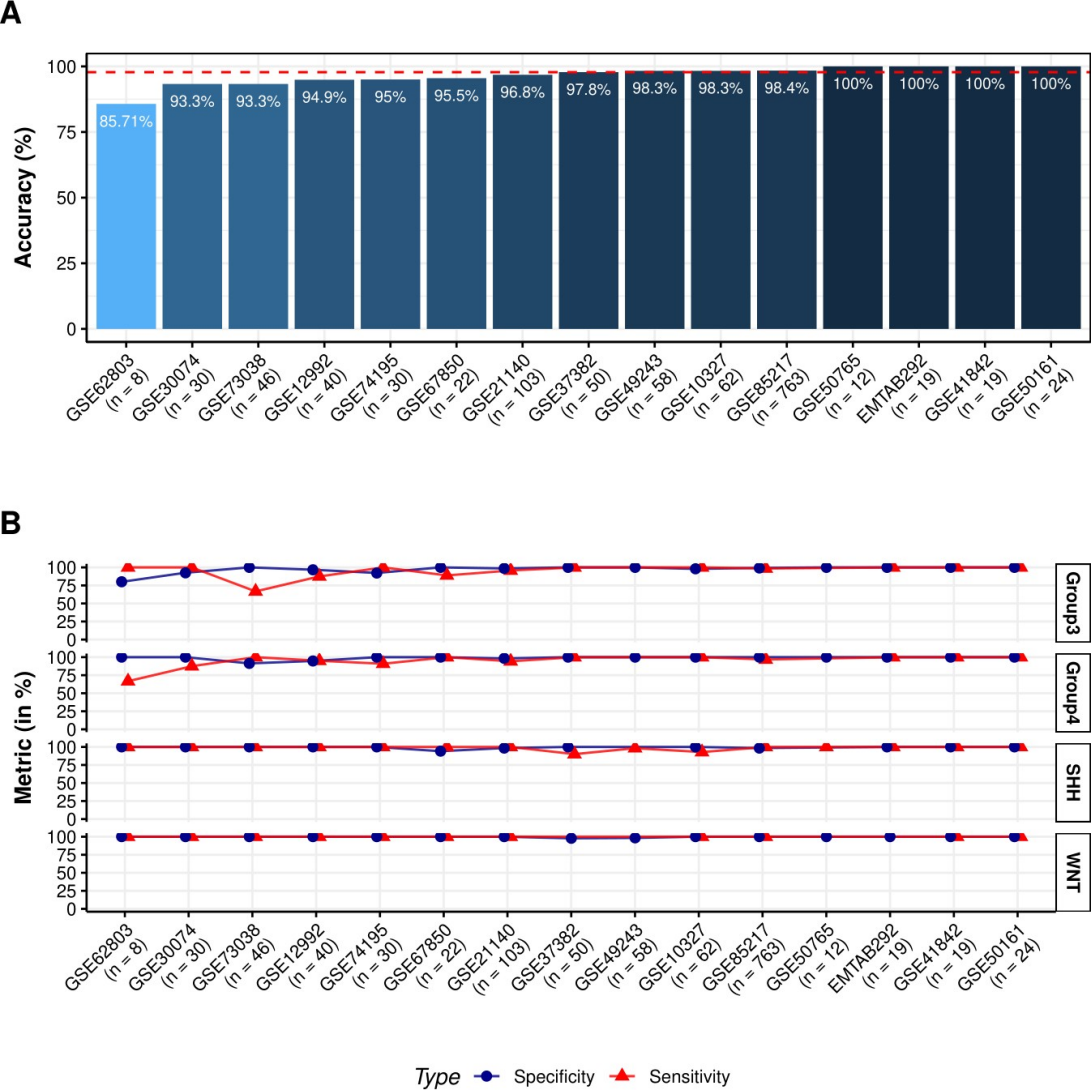
Classifier is able to distinguish SHH and WNT subtypes with higher accuracy than Group 3 and Group 4 subtypes. **A**. Bar Chart showing percent Accuracy of classification algorithm across 15 medulloblastoma microarray datasets. The dotted red line represents the median accuracy of 97.8% across all datasets. **B.** Line plot of Sensitivity and Specificity of classification algorithm trellised by molecular subtype across 15 medulloblastoma microarray datasets. On average, the classifier is able to classify SHH (Avg. Sensitivity: 98.7%; Avg. Specificity: 99.3%) and WNT (Avg. Sensitivity: 100%; Avg. Specificity: 99.7%) with better accuracy as compared to Group 3 (Avg. Sensitivity: 95.1%; Avg. Specificity: 97.1%) and Group 4 (Avg. Sensitivity: 94.7%; Avg. Specificity: 98.8%).

To test the classifier, we first applied it to the combined superset of 15 test datasets (N = 1,286 samples). The R package caret was used to obtain the overall confusion matrix, accuracy, and evaluation metrics, such as sensitivity and specificity, across the four subtypes (**Table 1**). Using this larger sample size, we observed an accuracy of 97.7%, sensitivity (TPR i.e. true positive rate) of 96.3%, 96.6%, 99.2% and 100% and specificity (TNR i.e. true negative rate) of 98.9%, 99.3%, 98.8% and 99.8% in predicting Group 3, Group 4, SSH and WNT subtypes, respectively. Next, the classifier was applied individually on each of the 15 test datasets to determine whether sample size had any effect on the classifier performance. We observed a minimum accuracy of 85.71% in the smallest dataset, GSE62803, which has a sample size of 8 and a maximum of 100% accuracy in four datasets (i.e. EMTAB292, GSE50765, GSE50161 and GSE41842). Overall, the classifier was able to differentiate between the four subtypes with a median accuracy of 97.8% across the 15 datasets (**Fig 5A**). On average across the 15 datasets, our classifier was able to maintain a specificity of 97.1%, 98.8%, 99.3% and 99.7% and sensitivity of 95.1%, 94.7%, 98.7% and 100% in predicting Group 3, Group 4, SSH and WNT subtypes, respectively (**Fig 5B**). Predictions across each of the 15 datasets and associated p-values are reported in **S3 Table** and summary stats are presented in **Table 2**.

**Table 1:** Confusion Matrix, Accuracy, and other evaluation metrics obtained after combining 15 test MB datasets followed by applying the classifier on the combined dataset (N = 1,286 samples).

Confusion Matrix				
	Ref_Group3	Ref_Group4	Ref_WNT	Ref_SHH
**Pred_Group3**	210	10	0	1
**Pred_Group4**	5	478	0	0
**Pred_WNT**	0	0	110	2
**Pred_SHH**	3	7	0	392
**Overall Stats**				
**Accuracy**	97.70%			
**Kappa**	96.70%			
**AccuracyLower**	96.70%			
**AccuracyUpper**	98.50%			
**AccuracyNull**	40.60%			
**AccuracyPValue**	0.00E+00			
**McnemarPValue**	NaN			
**Class Stats**				
	**Sensitivity**	**Specificity**	**Pos Pred Value**	**Neg Pred Value**
**Class: Group3**	96.30%	98.90%	95.00%	99.20%
**Class: Group4**	96.60%	99.30%	99%	97.70%
**Class: WNT**	100%	99.80%	98.20%	100%
**Class: SHH**	99.20%	98.80%	97.50%	99.60%
	**Precision**	**Recall**	**F1**	**Prevalence**
**Class: Group3**	95.00%	96.30%	95.70%	17.90%
**Class: Group4**	99%	96.60%	97.80%	40.64%
**Class: WNT**	98.20%	100%	99.10%	9.03%
**Class: SHH**	97.50%	99.20%	98.40%	32.43%
	**Detection Rate**	**Detection Prevalence**	**Balanced Accuracy**	
**Class: Group3**	17.24%	18.10%	97.60%	
**Class: Group4**	39.24%	39.70%	98%	
**Class: WNT**	9.03%	9.20%	99.90%	
**Class: SHH**	32.18%	33.00%	99.00%	

**Table 2:** Subtype-specific Sensitivity, Specificity and overall Accuracy across 15 test MB datasets.

Study	Sample_Size	Group3_Sensitivity	Group3_Specificity	Group4_Sensitivity		Group4_Specificity
**EMTAB292**	19	100%	100%	100%		100%
**GSE10327**	62	100%	98%	100%		100%
**GSE12992**	40	87.50%	96.80%	95%		94.70%
**GSE21140**	103	95.70%	98.60%	94.30%		98.30%
**GSE30074**	30	100%	92.60%		87.50%	100%
**GSE37382**	50	100%	100%	100%		100%
**GSE41842**	19	100%	100%	100%		100%
**GSE49243**	58	NA	100%	NA		100%
**GSE50161**	24	100%	100%	100%		100%
**GSE50765**	12	NA	100%	NA		100%
**GSE62803**	8	100%	80%	66.70%		100%
**GSE67850**	22	88.90%	100%	100%		100%
**GSE73038**	46	66.70%	100%	100%		91.40%
**GSE74195**	30	100%	92.30%	90.90%		100%
**GSE85217**	763	98.50%	99.30%	96.80%		100%
	**SHH_Sensitivity**	**SHH_Specificity**	**WNT_Sensitivity**	**WNT_Specificity**		**Accuracy**
**EMTAB292**	100%	100%	NA	100%		100%
**GSE10327**	92.90%	100%	100%	100%		98.30%
**GSE12992**	100%	100%	100%	100%		94.90%
**GSE21140**	100%	98.50%	100%	100%		96.80%
**GSE30074**	100%	100%	100%	100%		93.30%
**GSE37382**	90%	100%	NA	97.80%		97.80%
**GSE41842**	100%	100%	100%	100%		100%
**GSE49243**	98.30%	NA	NA	98.30%		98.30%
**GSE50161**	100%	100%	100%	100%		100%
**GSE50765**	100%	NA	NA	100%		100%
**GSE62803**	100%	100%	100%	100%		85.71%
**GSE67850**	100%	94.10%	100%	100%		95.50%
**GSE73038**	100%	100%	100%	100%		93.30%
**GSE74195**	100%	100%	100%	100%		95%
**GSE85217**	100%	98.50%	100%	100%		98.40%

Finally, we compared the MB classifier to an existing gene expression classifier for MB subtyping. The MB classifier outperformed the MM2S classifier for all 15 test datasets (**S3 Fig**). Although MM2S previously reported accuracies of 100% for WNT and SHH, 87.5% for Group 3, and 79.4% for Group 4 [23] for their dataset, when we applied the classifier to the 15 datasets herein, we observed combined accuracies of between 25–93.3%. Interestingly, the two datasets in which MM2S showed poorest performance (GSE62803: 25% and GSE74195: 38.5%) were skewed in favor of Group 3 and Group 4 subtypes. Our MB classifier achieved 85.7% and 95% for these datasets, respectively, indicating enhanced discrimination of these two subgroups with our MB classifier.

## Discussion

Here, we present a highly specific and sensitive GER-based medulloblastoma subtype classifier for use on transcriptomic data. All code related to the classifier development, training, and accuracy testing have been made available in github. We have also created an R package and corresponding R shiny application for easy accessibility to both the research community and clinical investigators. Additionally, we have licensed the software as GPL-3, which is Open Source Initiative compliant. Below are the direct links for the software:

Classifier development repository: https://github.com/d3b-center/medullo-classifier-dev

R package: https://github.com/d3b-center/medullo-classifier-package

Shiny web application code: https://github.com/d3b-center/medullo-classifier-shinyapp

Shiny web application: https://komalrathi.shinyapps.io/medulloclassifiershinyapp

Utilizing GERs, as opposed to traditional gene expression values, we were able to classify data stemming from orthogonal expression platforms and we demonstrate that this methodology is advantageous as a broad transcriptomic classifier. Specifically, the chosen GER features represent a union of features from DS1 and DS2 and this union could easily classify both DS1 and DS2 (i.e. GERs from DS2 did not decrease classification accuracy of DS1 and vice-versa). This lack of overlap between the two sets could be explained by the fact that the starting set of features was much larger (~100x) than the size of the reduced feature sets. Coupling this with the chosen cutoffs and other differences between the two datasets, such as platform, dynamic range, RNA degradation, we observed decreased overlap between the two sets.

Moreover, the set of GERs we discovered serves as our model, utilizing it with the very simple standardized score for classification. This allowed us to calculate the confidence in our call based on a *t-test* between the predicted class and the scores of the other classes. Despite other similar classification methods, including LASSO, the advantage of the GER solution is that it is relatively accessible, simple to use, and less susceptible to error when features are absent. Indeed, we demonstrate that utilizing GERs improved classification compared to k-nearest neighbor classification of a single sample gene-set enrichment analysis ranked matrix (MM2S).

We observed multiple WNT signaling genes within GERs associated with the WNT subtype. For example, *AXIN2* was the top-selected gene with 148 GERs associated with the signature [24]. The *LEF1* gene was the second most frequently selected gene with 40 representative GERs [25]. Additionally, *KREMEN1* was observed in GERs as a gene with high expression in the WNT subtype but low expression in the Groups 3 and 4 subtypes. Of note, the *NOTCH1*, *CTNNB1*, *WIF1*, *DLL3*, *DKK1*, *DKK2*, *DKK4*, *WNT11*, and *WNT16* signaling genes canonically over-expressed in the WNT subtype [7,8] did not arise as significant GERs. Many of these genes, while associated with the WNT pathway, are not differentially-expressed when compared to the other subtypes. For the nine genes listed above, only DKK2 and WIF1 were discriminatory for the formerly-named WNT subtype, subtype A, during classification by Kool, et. al. It is possible that gene expression is not the primary indicator of activity of these genes and further investigation should be performed. For instance, CTNNB1 nuclear localization may be a better marker of WNT pathway activity than its overall expression. We found that GERs containing *NEUROG1* (low), *GLI1* (high), *ATOH1* (high), and *OTX2* (low) were associated with the SHH signature. This aligns with a study from 2007 which reported high *ATOH1* and low *NEUROG1* expression samples with high *GLI1* expression, a biomarker of the SHH subtype [26] and a separate study which reported high *OTX2* expression in all subtypes except SHH [27]. Additional GERs for SHH contained the canonical over-expressed genes *HHIP*, *BOC*, and *SFRP1* (protein is an immunohistochemistry biomarker) [28], but not *SUFU*, *PTCH1*, or *MYCN*. As may be expected, the pathways commonly upregulated in WNT and SHH subtypes, cell cycle, NOTCH and PDGF pathways [8], were not represented in GERs in either subtype, as they are unable to discriminate these subtypes. Much less has been elucidated about discriminatory drivers, pathways, and markers of Group 3 and Group 4 MBs, but it is known that these tumors share some common pathways. For example, *PDGFRA*, which has low or no expression in Group 3 and 4 subtype tumors, was present in GERs for both subtypes. On the other hand, *LEMD1*, previously annotated as a specific marker for these subtypes [8], and *BLACAT1*, a non-coding RNA not previously associated with MB, were highly expressed and present in GERs for both subtypes. The *AFF3* gene could potentially discriminate the subgroups, as it was downregulated in Group 3, but upregulated in Group 4 tumors. Thus, the biological function of *BLACAT1* and *AFF3* in MB may be worth investigating. Specific to Group 4, we observed *CACNA1A* (*Calcium Voltage-gated Channel Subunit Alpha1 A*), in 21 GERs and was identified in systems biology analysis of Group 4 MBs as a novel therapeutic target [29]. Another recent study profiled the immune repertoire of MBs and found high expression of *CACNA1A* in Group 4 MB plasma cells [30]. *KHDRBS2* was present in 19 GERs and over-expressed in Group 4 tumors and was previously reported as a subgroup-specific gene [31]. Altogether, the classifier detected biomarkers and several canonical signaling pathway genes related to known MB subtypes. The lack of detection of other canonical genes might indicate their inability to discriminate subgroups as well as previously thought.

The MB classifier achieved high specificity and sensitivity across all datasets and the primary misclassifications came from Group 3 and Group 4 samples. As previously mentioned, these groups are known to have highly similar transcriptomic and genomic profiles which can lead to molecular ambiguity and histology misclassification. In fact, recent medulloblastoma genomic studies show extensive heterogeneity of the Group 3 and Group 4 subtypes and recommend splitting them into as many as 8 subtypes [18]. It should be noted that patients who present tumors of these molecular subtypes are currently put on the same treatment regimen [5, 12], so previous misclassification of these groups has not affected treatment recommendations to date, however, ongoing studies are attempting to stratify treatment by molecular subtype, highlighting the need for accurate subtyping. Nevertheless, potential misclassification may lead to discrepancies in prognosis, therefore, additional classification approaches are recommended. Future directions include evaluating ways to further reduce the features set without compromising sensitivity and specificity using standard pruning methods. Additionally, we plan to update our current approach of calculating a p-value to establish confidence in a class to a more refined calibration method such as histogram binning or Bayesian Binning into Quantiles [32]. Finally, we intend to apply this classification method to additional underexplored pediatric and adult cancer types. Creating additional cancer classification applications has the potential to enable fast, simple, and accurate RNA-based subtyping from clinical samples, thereby accelerating subtyping and treatment regimens.

## Supporting information

S1 Fig Genes with high expression and high variability reduce noise in feature selection. **A.** Distribution of maximum FPKM across 97 primary medulloblastoma samples (RNA-seq: EGAD00001001899). **B.** Distribution of standardized CVs per gene across 97 primary medulloblastoma samples (RNA-seq: EGAD00001001899).
(TIF)

S2 Fig GERs show a normal distribution which is similar and comparable across different datasets. **A.** Distribution of maximum GERs across 97 primary medulloblastoma samples (RNA-seq: EGAD00001001899). **B.** Distribution of maximum GERs across 76 primary medulloblastoma samples (Microarray: GSE37418).
(TIF)

S3 Fig Accuracy benchmarking of MB classifier vs. MM2S. Barplot of percent accuracy comparison of MB classifier with MM2S using 15 test microarray datasets shows MB classifier performs better than MM2S in every case. Dotted lines represent the median accuracies across all datasets for the MB classifier and MM2S classifier.
(TIF)

S1 Table 1,399 genes associated with specific medulloblastoma subtypes. (CSV)

S2 Table 1,795 GERs associated with specific medulloblastoma subtypes. (CSV)

S3 Table Subtype classification with p-value significance (t-test) of each sample across 15 test MB datasets. (XLSX)
