# Supplementary figures and images for: A transcriptome-based classifier to determine molecular subtypes in medulloblastoma
Source: PLoS Comput Biol. 2020 Oct 29;16(10):e1008263. doi: 10.1371/journal.pcbi.1008263 (PMC7654754; doi:10.1371/journal.pcbi.1008263)

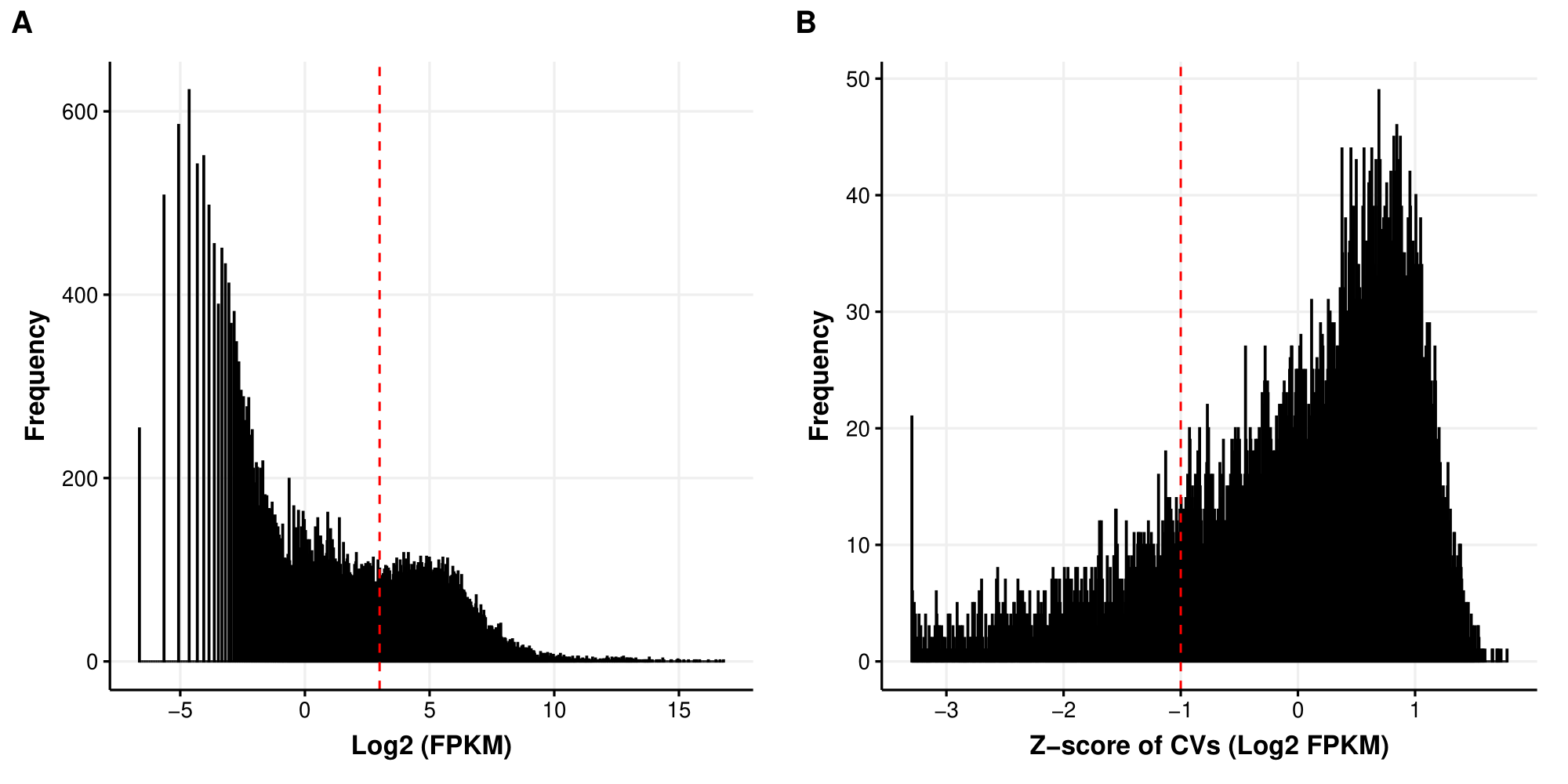

Supplement: S1 Fig — A. Distribution of maximum FPKM across 97 primary medulloblastoma samples (RNA-seq: EGAD00001001899). B. Distribution of standardized CVs per gene across 97 primary medulloblastoma samples (RNA-seq: EGAD00001001899). (TIF) [file pcbi.1008263.s001.tif]

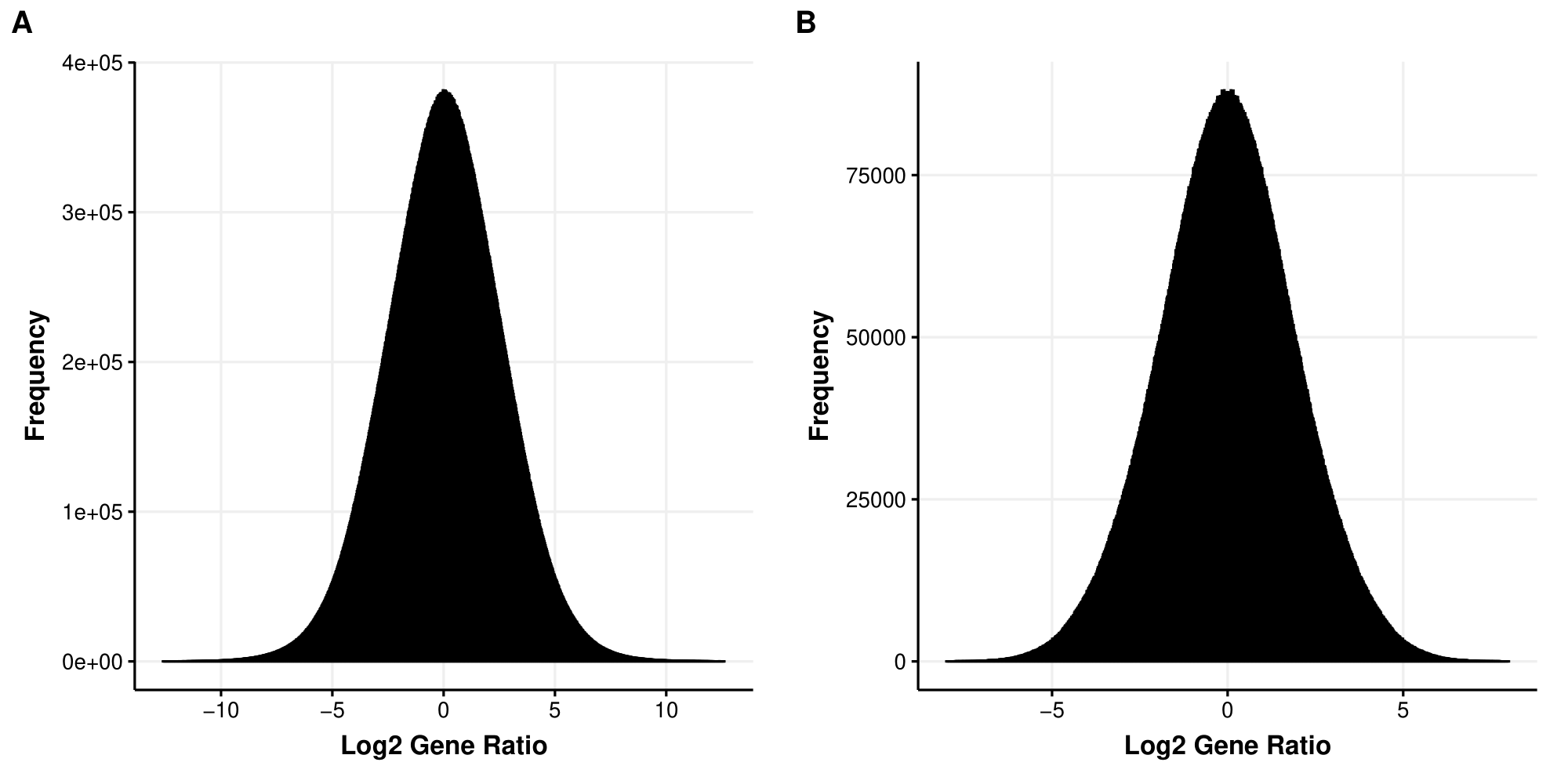

Supplement: S2 Fig — A. Distribution of maximum GERs across 97 primary medulloblastoma samples (RNA-seq: EGAD00001001899). B. Distribution of maximum GERs across 76 primary medulloblastoma samples (Microarray: GSE37418). (TIF) [file pcbi.1008263.s002.tif]

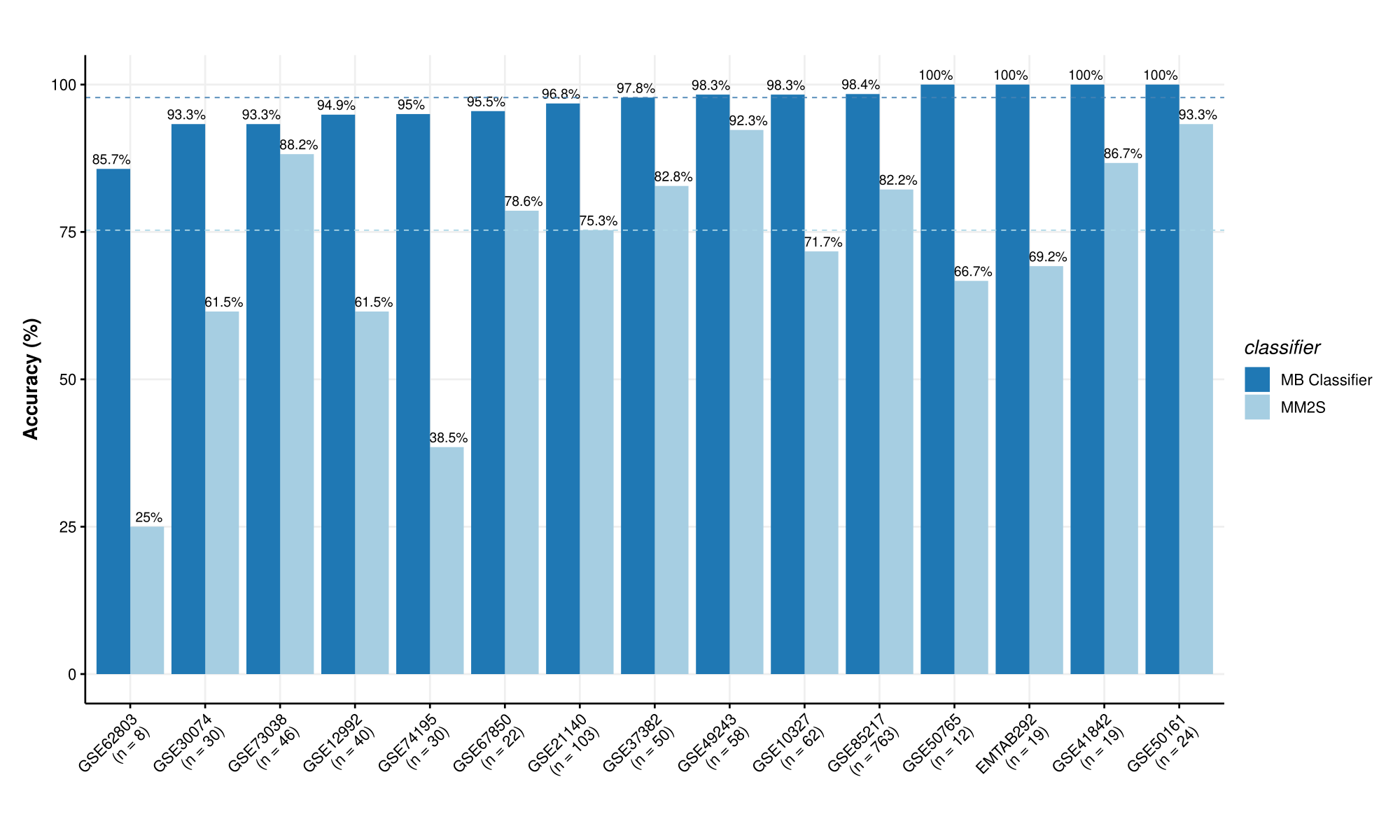

Supplement: S3 Fig — Barplot of percent accuracy comparison of MB classifier with MM2S using 15 test microarray datasets shows MB classifier performs better than MM2S in every case. Dotted lines represent the median accuracies across all datasets for the MB classifier and MM2S classifier. (TIF) [file pcbi.1008263.s003.tif]
